# Investigations on Metabolic Changes in Beagle Dogs Fed Probiotic Queso Blanco Cheese and Identification of Candidate Probiotic Fecal Biomarkers Using Metabolomics Approaches

**DOI:** 10.3390/metabo10080305

**Published:** 2020-07-25

**Authors:** Ye Jin Kim, Ho-Eun Park, Wan-Kyu Lee, Jun-Sang Ham, Sang Un Park, Jae Geun Kim, Kyung-Hoan Im, Jae Kwang Kim

**Affiliations:** 1 Division of Life Sciences, College of Life Sciences and Bioengineering, Incheon National University, Yeonsugu, Incheon 22012, Korea; 201721047@inu.ac.kr (Y.J.K.); khim61@inu.ac.kr (K.-H.I.); 2 College of Veterinary Medicine, Chungbuk National University, Cheongju 28644, Korea; phu4793@naver.com (H.-E.P.); wklee@chungbuk.ac.kr (W.-K.L.); 3 Animal Products Development and Utilization Division, National Institute of Animal Science, Wanju 55365, Korea; hamjs@korea.kr; 4 Department of Crop Science, Chungnam National University, 99 Daehak-ro, Yuseong-gu, Daejeon 34134, Korea; supark@cnu.ac.kr

**Keywords:** fecal metabolic profiling, probiotic cheese, *Bifidobacterium longum* KACC 91563, *Lactobacillus reuteri* KACC 92293, dog

## Abstract

Intake of probiotic cheese improves the intestinal health of humans and animals. However, metabolic changes in the intestines of dogs in response to the ingestion of probiotic cheese have not been evaluated. Thus, we aimed to determine the metabolic changes in healthy beagle dogs fed queso blanco cheese with added *Lactobacillus reuteri* KACC 92293 and *Bifidobacterium longum* KACC 91563 (QCLB) and to identify potential fecal biomarkers to distinguish the metabolic changes based on intake of probiotic cheese through metabolomics approaches. The dogs were randomly divided into three groups and fed a regular diet without any cheese (control), a diet with queso blanco cheese (QC), or one with QCLB for eight weeks. The concentrations of acetic, propionic, and 4-aminobutyric acids were increased in the QCLB group compared to those in the control group. Additionally, higher levels of propionic acid and lower levels of xylose were found in the QCLB group compared to those in the QC group. This is the first report on the identification of metabolic changes in beagle dogs fed queso blanco cheese with added *L. reuteri* KACC 92293 and *B. longum* KACC 91563. We also found that metabolomics approaches can be useful for identifying potential fecal markers in dogs fed probiotic cheese.

## 1. Introduction

Probiotics are living microorganisms that have beneficial effects on intestinal health. They are associated with better fecal consistency and reduced infections and inflammatory bowel disease in animals and humans [1,2]. Lactic acid bacteria, such as lactobacilli and bifidobacteria species, are the most commonly used probiotics [3]. One species of lactobacillus, *Lactobacillus reuteri*, has been the focus of numerous studies because it is known to colonize several regions of the body, such as the skin and gastrointestinal and urinary tracts, in many mammals. *L. reuteri* confers health benefits on the host and helps regulate immune responses and reduce infections [4,5]. *Bifidobacterium longum* is one of the ten most common species of *Bifidobacterium* [6]. *B. longum* ssp. is used for probiotic therapy in chronic inflammatory bowel disease. [7,8]. Along with research into the use of single probiotics, studies on the use of multiple probiotics strains simultaneously have been performed because various probiotics can enhance the protective effects of several pathogens in the intestine [9].

Because of their known benefits, probiotics are added to many dairy foods. Among probiotic foods, cheese is a good medium for delivering probiotics into the intestine because of its generally higher pH and fat levels than milks [10]. In humans, probiotic cheese ingestion increases *L. rhamnosus* HN001 and *L. acidophilus* ATCC 700396 numbers in feces [11]. Recently, studies on the effects of probiotic supplements in dogs have attracted attention because the gut microbiota of dogs resembles the human gut microbiota more closely than those of pigs and mice [12]. In such studies, including those in which intestinal microbiota were analyzed, it has been observed that supplementation of probiotics reduces the abundance of many pathogenic bacteria, such as *Clostridium perfringens* and *Stenotrophomonas maltophilia*, in the intestines of dogs [13]. In addition, ingested probiotics change not only the gut microbiota but also fecal metabolites. Thus, profiling of fecal metabolites in dogs has the potential to provide more comprehensive information regarding the metabolic changes induced by the ingestion of probiotic foods by animals and humans [14].

Metabolomics can reveal metabolic changes of small-molecule metabolites (< 1500 Da) such as amino acids, organic acids, and volatiles in biological fluids. Several techniques, such as gas chromatography–mass spectrometry (GC–MS), GC-flame ionization detection (FID), liquid chromatography (LC)–MS, and capillary electrophoresis (CE)-MS, have been mainly used for the selective and sensitive analysis of metabolites in biological samples [14]. Among these techniques, GC–MS is the preferred one, considering its relatively fast screening and inexpensive analytical system [15,16]. GC–FID exhibits selectivity and specificity for fatty acids [17]. Metabolomic approaches are now widely used to identify biomarkers for the characterization and diagnosis of many diseases [18]. The technique is comprehensive and can be quantitative through the use of metabolic profiling with multivariate analysis [19]. Metabolomics have been used to investigate how ingested probiotics affect metabolic changes in the intestinal environments of humans and mice [20]. We previously confirmed increased levels of probiotics and short chain fatty acids in the feces of dogs after ingestion of queso blanco cheese containing *B. longum* KACC 91563 [21]. We also previously demonstrated that administration of queso blanco cheese containing *B. longum* KACC 91563 has a positive effect on the fecal microbiota population and immune response in beagle dogs by analysis of fecal microbiota and serum cytokines [22]. However, there is still insufficient information on intestinal metabolic changes induced by ingestion of cheese containing various probiotics.

In this study, we used metabolic approaches to investigate the effect of queso blanco cheese with added *L. reuteri* KACC 92293 and *B. longum* KACC 91563 (QCLB) by conducting metabolic profiling with GC–MS and GC–FID. Furthermore, we aimed to identify potential fecal markers to determine any improvements in dogs fed probiotic cheese using multivariate statistics.

## 2. Results and Discussion

### 2.1. Comparison of Fecal Metabolic Profiles between the Three Dietary Groups

Probiotic foods have potential effects on intestinal metabolism associated with fatty acids, amino acids, and sugars [23]. Thus, in our study, fecal metabolic profiling was performed to explore the metabolic changes in feces of dogs fed probiotic cheese containing *L. reuteri* KACC 92293 and *B. longum* KACC 91563. We used GC–MS and GC–FID for the rapid and sensitive screening of metabolites. A total of 55 metabolites (i.e., Metabolomics Standards Initiative (MSI) level 1), including five volatile fatty acids (VFAs), one indolic compound, 15 long chain fatty acids (LCFAs), 16 amino acids, 10 organic acids, six sugars, and two sugar alcohols were identified (Supplementary materials Tables S1–S3).

**Figure 1:**
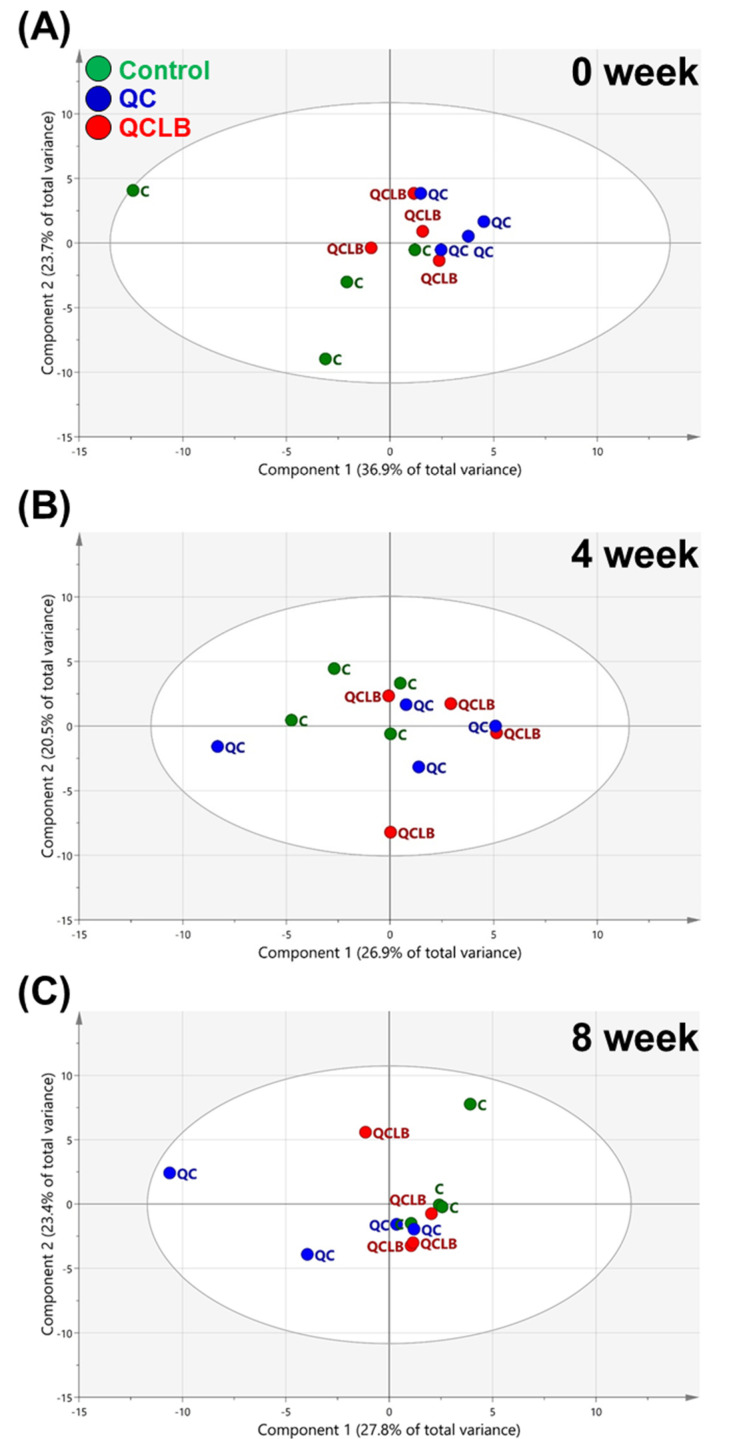
Score plots of the principal component analysis (PCA) model derived from 55 metabolites in the feces of beagle dogs fed a control, QC, or QCLB diet for 0 (**A**), 4 (**B**), and 8 (**C**) weeks. Control, regular diet without any cheese; QC, regular diet and queso blanco cheese; QCLB, regular diet and queso blanco cheese with added *L. reuteri* KACC 92293 and *B. longum* KACC 91563.

**Figure 2:**
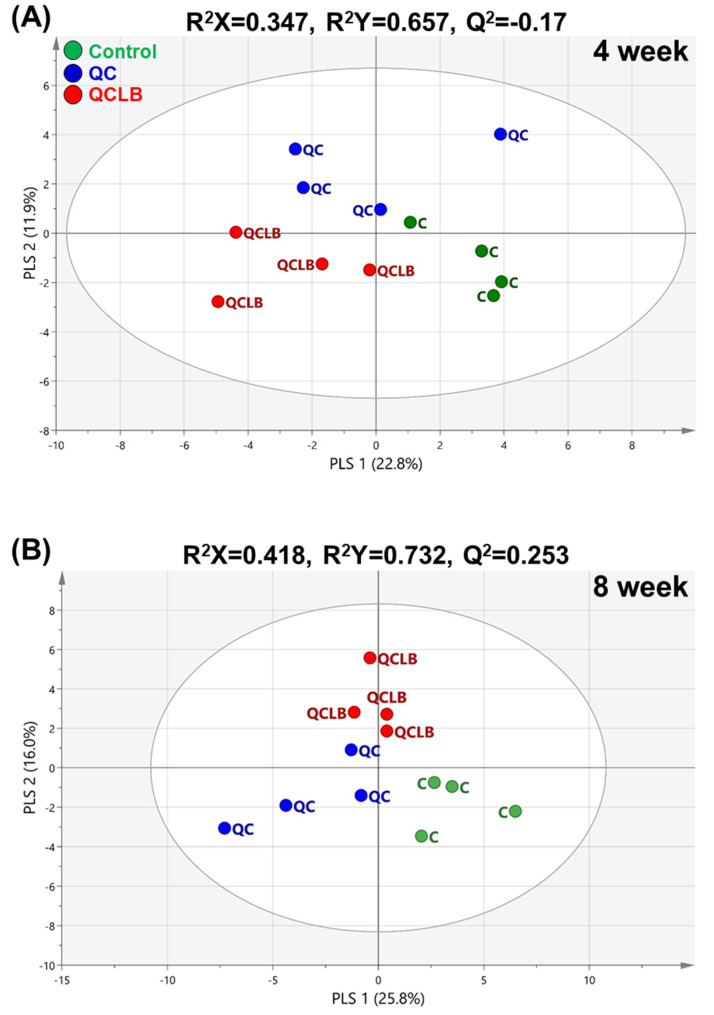
Score plots of the partial least squares-discriminant analysis (PLS-DA) model derived from 55 metabolites in the feces of beagle dogs fed a control, QC, or QCLB diet for 4 (**A**) and 8 (**B**) weeks. Control, regular diet without any cheese; QC, regular diet and queso blanco cheese; QCLB, regular diet and queso blanco cheese with added *L. reuteri* KACC 92293 and *B. longum* KACC 91563.

The three groups were not separated in the principal component analyses (PCA) score plot at week 0 (Figure 1A), suggesting that there was no difference in the physical condition of individuals in each group before the start of the experiment. Additionally, no major differences were seen between the three groups at weeks 4 and 8 (Figure 1B,C). Therefore, partial least-squares discriminant analyses (PLS-DA) was conducted to evaluate potential differences in fecal metabolites at weeks 4 and 8. The PLS-DA was able to classify the control, QC, and QCLB groups. After four weeks, the PLS-DA model of the three groups had an R^2^Y value of 0.657 and Q^2^ value of −0.17. The low Q^2^ value suggests that there was no separation between the different diets (Figure 2A). However, the PLS-DA model at eight weeks had an R^2^Y value of 0.732 and Q^2^ value of 0.253 (Figure 2B). Although the Q^2^ value was lower than 0.5, more of a difference between the three groups was seen in the score plot at week 8 than at week 4. In other words, fecal metabolites of beagle dogs were affected differently by the control, QC, and QCLB diets.

**Figure 3:**
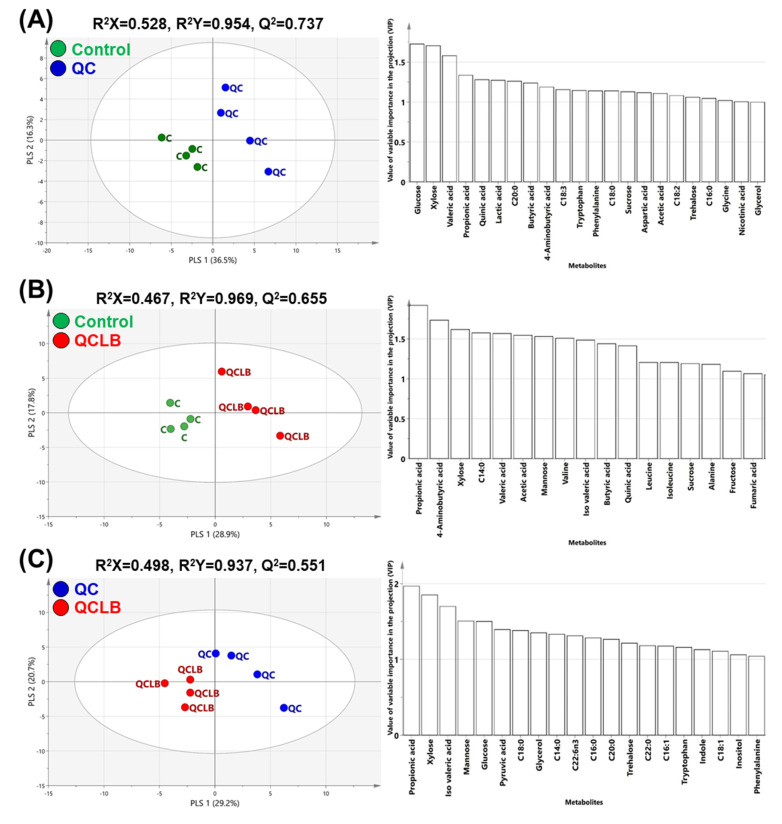
Score (left) and the variables important in the projection (VIP, right) plots of the PLS-DA models obtained from 55 metabolites in the feces of beagle dogs fed a control, QC, or QCLB diet for 8 weeks. PLS-DA models indicate regressions between the control and QC (**A**), QCLB and control (**B**), or QCLB and QC (**C**) groups. Metabolites that had VIP-values > 1.0 are indicated in the VIP plots. C14:0, myristic acid; C16:0, palmitic acid; C16:1, palmitoleic acid; C18:0, stearic acid; C18:1, oleic acid; C18:2, linoleic acid; C18:3, linolenic acid; C20:0, arachidic acid; C22:0, behenic acid. control, regular diet without any cheese; QC, regular diet and queso blanco cheese; QCLB, regular diet and queso blanco cheese with added *L. reuteri* KACC 92293 and *B. longum* KACC 91563.

After eight weeks, the three paired PLS-DA models had R^2^Y values of 0.937–0.969 and Q^2^ values of 0.551–0.737 and the control, QC, and QCLB groups clustered separately in the score plots of each PLS-DA model (Figure 3). The highest the variables important in the projection (VIP) values in the control and QC groups represented sugars and VFAs such as glucose, xylose, propionic acid, and valeric acid (Figure 3A). Additionally, sugars, fatty acids, and amino acids, including propionic acid, 4-aminobutyric acid (GABA), xylose, C14:0, valeric acid, acetic acid, mannose, valine, and isovaleric acid, were found as important metabolites in the VIP plot of the QCLB and control groups (Figure 3B). Propionic acid, xylose, isovaleric acid, and mannose were identified as the highest contributors in the PLS-DA model comparing the QCLB and QC groups (Figure 3C).

### 2.2. Identification of Potential Fecal Biomarkers Related to Metabolic Changes Induced by Probiotic Cheese Ingestion in Beagle Dogs

**Figure 4:**
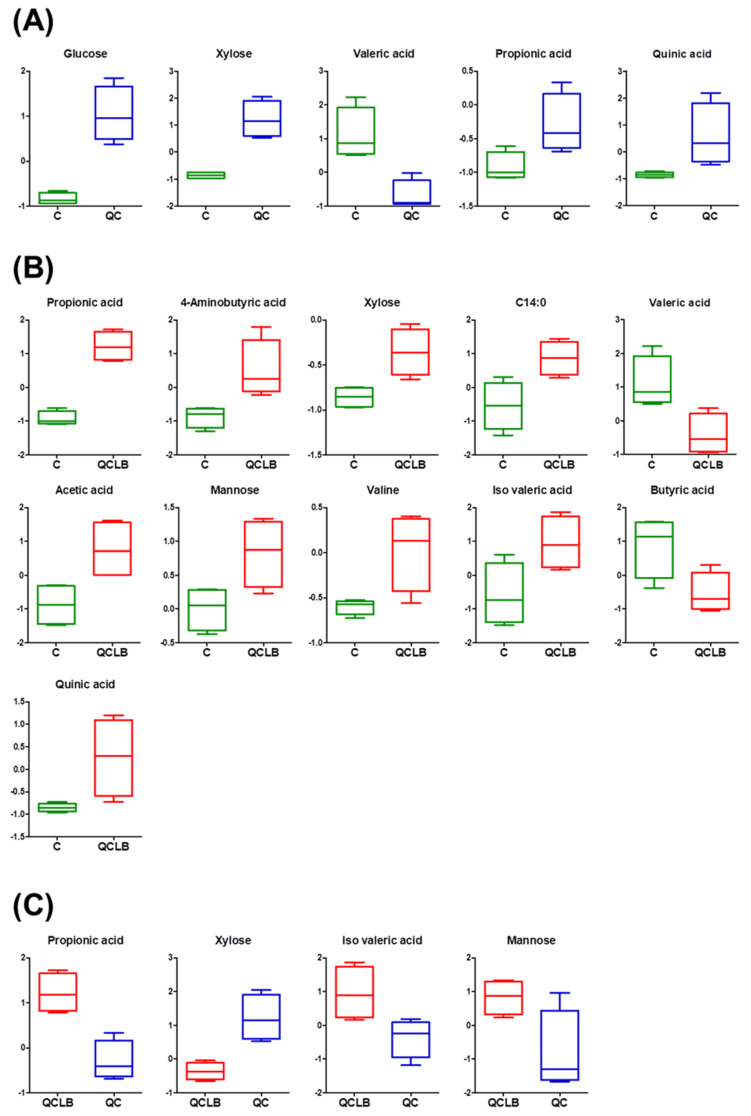
Metabolites that differed significantly (*p* < 0.05) in the feces of beagle dogs between the control and QC (**A**), QCLB and control (**B**), or QCLB and QC (**C**) groups after 8 weeks. C, control; C14:0, myristic acid; QC, regular diet and queso blanco cheese; QCLB, regular diet and queso blanco cheese with added *L. reuteri* KACC 92293 and *B. longum* KACC 91563.

Metabolic changes induced by the three diets were identified (Figure 4). Before comparing the changes in the fecal metabolites among the three groups of beagle dogs fed different diets for eight weeks, the effect of gender was examined for each group using a dendrogram for clustering analysis; we did not observe a gender-based difference in any of the groups (Figure S1). First, in the comparison between the control and QC groups, five metabolites (glucose, xylose, propionic acid, valeric acid, and quinic acid) were identified as significant (Figure 4A). Among these metabolites, glucose, xylose, and propionic acid showed increases in the QC group. Queso blanco cheese has high carbohydrate, protein, and fat contents [24]. The levels of carbohydrates in the cheese are not detrimental to health, and are fermented by probiotics to form monosaccharaides such as glucose, fructose, xylose, and mannose, which help to produce VFAs using carbohydrate transporters [25]. Additionally, because cheese creates a favorable environment for probiotics, *Lactobacillus* spp. used as a probiotic survives in most cheeses and produces VFAs, which can reduce the risk of developing gastrointestinal disorders [26]. Thus, finding glucose, xylose, and propionic acid in dog feces fed the QC diet suggests that intake of queso blanco cheese increases probiotics in the intestine that produce propionic acid using monosaccharaides. Queso blanco cheese therefore appears to help create a favorable environment for the proliferation of probiotics in the intestine.

In the comparison between the QCLB and control groups, there were significant differences in 11 metabolites (Figure 4B). Higher levels of xylose, mannose, valine, and GABA were detected in the feces of dogs fed the QCLB diet. Xylose, mannose, and valine are related to the process of fermentation by the intestinal microbiota of carbohydrates and proteins from the probiotic cheese. Additionally, as a non-protein amino acid, GABA improves symptoms in gastrointestinal disorders such as irritable bowel syndrome and constipation [27]. *Lactobacillus* and *Bifidobacterium* species grown in probiotic cheeses induce higher synthesis of GABA than other dairy products [28]. High levels of propionic acid, acetic acid, and isovaleric acid were detected in the QCLB group, while butyric and valeric acids were increased in the control group. Isovaleric acid derived from branched-chain amino acid is a representative aromatic compound of cheese and is produced more in probiotic cheese than in probiotic cultures [29,30]. Butyric and valeric acids are produced by harmful bacteria such as *Clostridium* species [22,31]. On the other hand, propionic and acetic acids are related to probiotics such as lactobacilli and bifidobacteria. Consequently, levels of the beneficial metabolites GABA, acetic acid, and propionic acid were increased by ingestion of probiotic cheese. Based on these results, we propose that ingestion of QCLB has a positive influence on the intestine of dogs compared to the ingestion of a regular diet.

In the comparison between the QCLB and QC groups, there were significant differences in four metabolites: propionic acid, isovaleric acid, xylose, and mannose (Figure 4C). The comparison between the QCLB and QC groups was performed to evaluate the metabolic changes induced by *L. reuteri* KACC 92293 and *B. longum* KACC 91563, because the difference between these two groups was the addition of probiotics in the queso blanco cheese. Among these metabolites, propionic acid was increased, and xylose was decreased in feces fed the QCLB diet. It has been reported that *L. reuteri* administration increases levels of propionic acid and improves the gut health of neonatal piglets [32]. Both *L. reuteri* and *B. longum* are known to produce VFAs such as acetic, propionic, and butyric acids from xylose and glucose and fructose [33,34]. Therefore, the metabolic changes on propionic acid and xylose in the intestine of the QCLB group result from increases in the number of *L. reuteri* KACC 92293 and *B. longum* KACC 91563 from the QCLB diet. These results further confirm the probiotic changes of queso blanco cheese with added *L. reuteri* KACC 92293 and *B. longum* KACC 91563 in dog intestines. In order to discriminate the differences of feces metabolites in dogs affected by the probiotic cheese, we suggest propionic acid and xylose as candidate fecal biomarkers.

### 2.3. Visualization of Dynamic Metabolic Changes in Feces after Probiotic Cheese Ingestion

**Figure 5:**
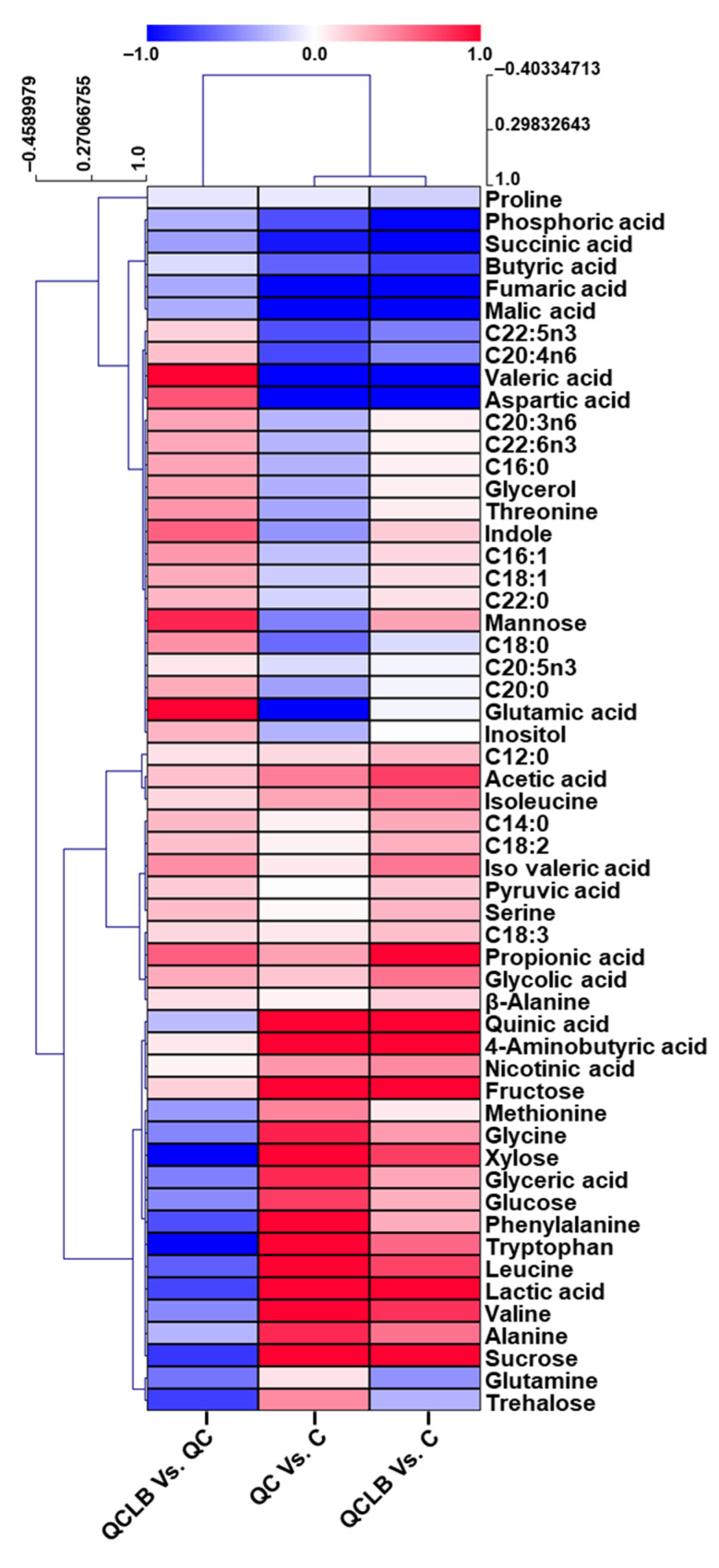
Hierarchical clustering heat map of log2FC values obtained from 55 fecal metabolites in comparisons between pairs of groups. The three paired comparisons were QC Vs. control, QCLB Vs. control, and QCLB Vs. QC and are indicated on the x-axis while the 55 metabolites are indicated on the y-axis. The up and down-regulated metabolites are expressed with red and blue, respectively. Red shading represents a log2FC value of > 0, while blue shading represents log2FC < 0. C, control; C12:0, lauric acid; C14:0, myristic acid; C16:0, palmitic acid; C16:1, palmitoleic acid; C18:0, stearic acid; C18:1, oleic acid; C18:2, linoleic acid; C18:3, linolenic acid; C20:0, arachidic acid; C22:0, behenic acid; QC, regular diet and queso blanco cheese; QCLB, regular diet and queso blanco cheese with added L. reuteri KACC 92293 and B. longum KACC 91563.

Our hierarchical clustering heat map of log_2_FC values show the variations of fecal metabolites after probiotic cheese ingestion compared to the regular cheese diet or regular diet without cheese for eight weeks (Figure 5). In the heat map, VFAs and sugars except for mannose, butyric acid, and valeric acid were clustered. These metabolites clearly show the differences in metabolic changes of the three comparisons after eight weeks of feeding. The highest levels of acetic acid and propionic acid were found in the QCLB group. Monosaccharides such as glucose, fructose, and xylose were lower in the comparison between the QCLB and QC groups than in the other comparisons. These results support the probiotic effect of the QCLB diet that produces higher levels of acetic and propionic acids derived from monosaccharides. The detailed visualization provided by the hierarchical clustering heat map of log_2_FC effectively reflects the dynamic changes of individual fecal metabolites.

## 3. Materials and Methods

### 3.1. Preparation of Cheese

Queso blanco cheese (QC) was made according to Ham et al. [31] with some modifications. Four hundred grams of citric acid in 20 L of water was added to 100 kg of skimmed milk, with no addition of salt. *L. reuteri* KACC 92293 and *B. longum* KACC 91563 for the QCLB were added before molding. Each cheese was stored at 4 °C and fed for 8 weeks (QC moisture 56.5–57.5%, protein 34.3–35.2%, lactic acid bacterial counts 3.58–4.02 log CFU/g; QCLB moisture 53.8–55.0%, protein 36.1–36.4%, lactic acid bacterial counts 5.88–6.47 log CFU/g, bifidobacteria counts 6.62–7.86 log CFU/g).

### 3.2. Animals and Experimental Design

Twelve healthy beagle dogs (6 females and 6 males) were used in the trial. The body weight range of the dogs was 8–12 kg. One beagle dog was housed per cage (W60 × L70 × W80 cm^3^). The animals were reared at a constant temperature of 21 °C ± 2 °C (69.8 °F ± 35.6 °F), humidity of 30% to 70%, ventilation of 14 to 18 air changes/h, light duration of 12 hours, and light level of 150 to 300 Lux, in accordance with conditions specified by the Chungbuk National University Laboratory Animal Research Center. Each experimental animal received 250 g of feed per day for laboratory dogs (Cargill Agri Purina Inc., Sungnam, South Korea). The experimental dogs were randomly divided into three groups: control (three males, one female), QCLB (one male, three females), and QC (two males, two females). The QCLB group was fed 10 g/kg body weight of queso blanco cheese containing *L. reuteri* KACC 92293 and *B. longum* KACC 91563 for 8 weeks. The QC group was fed queso blanco cheese devoid of *L. reuteri* KACC 92293 and *B. longum* KACC 91563. A control group was not fed any cheese. The experimental period was from 7/2/2018 to 8/27/2018. Fresh fecal samples were collected 3 times, before intake of cheese (week 0, 7/2/2018), during cheese intake (week 4, 7/30/2018), and after cheese intake (week 8, 8/27/2018), respectively. The study was approved by the Institutional Animal Care and Use Committee of Chungbuk National University (approval number CBNUA-1080-18-02). The collected fecal samples were stored at −80 °C until further analyses.

### 3.3. Analysis of Volatile Fatty Acids and Indolic Compounds in Feces of Healthy Beagle Dogs

The volatile fatty acids (VFAs) and indolic compounds in feces were analyzed using headspace solid-phase microextraction (HS-SPME) with GC–MS, based on previously described protocols [22]. Feces samples (50 mg) were blended with 280 μL of distilled water, 10 μL of 2-methylvaleric acid (internal standard (IS), 1 mg/mL), and 30 μL of 0.9 M sulfuric acid in a 10 mL crimp vial (5182-0838, Agilent Technologies, CA, USA) containing a magnetic stirring bar (size: 7 × 2 × 2 mm). The vial was sealed using an aluminum cap with a polytetrafluoroethylene/silicone septum (226-84525-11, Shimadzu, Tokyo, Japan) and was put on a hot plate for 60 min at 60 °C and 20× *g*. A SPME fiber (50/30 μm divinylbenzene/carboxen/polydimethylsiloxane (DVB/CAR/PDMS, 2 cm), 57348-U, Supelco, Bellefonte, PA, USA) was equipped with a SPME fiber holder (57330-U, Supelco, Bellefonte, PA, USA) and rinsed using SPME fiber conditioner (Field forensics, St. Petersburg, FL, USA) for 20 min at 250 °C. The pre-conditioning fiber was inserted into the sample vial. The volatile compounds were concentrated for 15 min at 60 °C and 5× *g*. After concentration, the fiber was injected into an injector pole.

A GC–MS analysis was conducted using a Shimadzu GCMS-QP2010 Ultra system (Shimadzu, Kyoto, Japan), equipped with a DB-5 column (0.25 mm × 0.25 μm × 30 m, 122-5033, Agilent, Santa Clara, CA, USA). The parameters were set as follows: split (20:1), 250 °C injector temperature, carrier gas (helium) constant flow of 1.3 mL/min, 260 °C interface temperature, and 250 °C ion source temperature. The oven program was as follows: 60 °C held for 3 min and then increased at 40 °C/min to 260 °C, then maintained for 5 min. The MS scan region was 30–250 *m*/*z*. The selected ion monitoring mode was performed at 43 *m*/*z* for acetic acid; at 60 *m*/*z* for butyric, valeric, and isovaleric acid; at 74 *m*/*z* for propionic and 2-methylvaleric acid; and at 117 *m*/*z* for indoles. Data acquisition and processing were carried out using LabSolutions GC–MS software version 4.11 (Shimadzu, Kyoto, Japan). Peak identifications were compared with retention time and mass spectra fragmentation of standards. For quantitative analysis of volatile compounds, the analyte concentration as the ratio of the compound peak area to IS peak area was proportionally calculated with a standard concentration.

### 3.4. Analysis of Long Chain Fatty Acids in Feces of Healthy Beagle Dogs

Analysis of long chain fatty acids (LCFAs) in feces was performed using previously described methods [35]. In total, 2.5 mL of chloroform:methanol (2:1, *v*/*v*) solution and 100 μL of pentadecanoic acid (IS, 1 mg/mL) were mixed with each fecal sample (100 mg) per 15 mL tube. The mixture was sonicated for 20 min. After sonication, 2.5 mL of 0.58% sodium chloride in distilled water was added to the tube. The tube was vortexed for 20 s and then centrifuged at 15,000× *g* and 4 °C for 5 min. The chloroform phase (under layer) was separated to a new tube and then dried using a speed-vac concentrator (VS-802F, Visionbionex, Gyeonggi, Korea). The concentrated sample was methylated with 180 μL of methanol, 100 μL of toluene, and 20 μL of 5 M sodium hydroxide at 85 °C and 300 rpm for 5 min. Then, 300 μL of boron trifluoride was added to the mixture and reacted under the same conditions. After incubation, 400 μL of distilled water and 800 μL of pentane were added. The mixture was blended for 20 s and centrifuged at 350× *g* and 4 °C for 15 min. The supernatant was transferred into a fresh 2 mL tube. The dried sample was dissolved in 100 μL of hexane. The sample was filtered through a 0.5 μm syringe filter and analyzed by GC–FID. The GC system consisted of an Agilent 7890A gas chromatograph equipped with a DB-WAX column (30 m × 0.25 mm × 0.25 μm, 122-7032, Agilent, Santa Clara, CA, USA) and 7890 GC detector. The flow rate of the carrier gas (nitrogen) was 1 mL/min. The front inlet and detector temperatures were set at 250 °C. The column temperature conditions were as follows: the initial temperature was maintained at 130 °C for 3 min; then it was raised to 230 °C at a rate of 20 °C/min; and the final temperature was increased to 250 °C at a rate of 3 °C/min and maintained for 5 min. Qualitative and quantitative analyses of LCFAs were conducted using saturated and unsaturated fatty acid methyl ester (FAME) mixes (CRM18918, Supelco, Bellefonte, PA, USA).

### 3.5. Analysis of Hydrophilic Compounds in Feces of Healthy Beagle Dogs

Previously described methods were used to extract the hydrophilic compounds in feces of health beagle dogs [36]. One milliliter of methanol:water (8:2, *v*/*v*) solution and 30 μL of L-2-chlorophenylalanine as an IS (0.3 mg/mL) were sonicated with each sample (100 mg) for 15 min. After sonication, the mixture was centrifuged at 14,000× *g*, for 15 min at 4 °C. The supernatant was separated and transferred to a clean tube and dried using a centrifugal concentrator for ~3 h. To derivatize the sample, 80 μL of methoxylamine hydrochloride (MOX, 20 mg/mL) was added and reacted by mixing at 1200 rpm for 90 min at 37 °C. The sample was then mixed with 80 μL of N-O-bis-(trimethylsilyl)-trifluoroacetamide + trimethylchlorosilane (BSTFA + 1% TMCS) and heated again at 60 °C and 1200 rpm for 60 min. Finally, the sample was analyzed by GC–MS. The GC–MS system was configured identically to the analysis of volatile compounds. Split injection was conducted with a split ratio of 1:10. The injector temperature was set at 280 °C. The column oven temperature was 100 °C held for 4 min; then increased at a rate of 10 °C/min to 320 °C; and finally, held at 320 °C for 11 min. The interface and ion source temperatures were maintained at 280 and 200 °C, respectively. The carrier gas was helium, and the flow rate was 1.1 mL/min. The mass range was scanned from 45 to 600 amu. The hydrophilic compounds were identified by comparing their retention times and mass fragmentation patterns with reference to standard compounds from the in-house library and MS library (Nist and Wiley 9). Quantitative determinations were carried out based on the ratio of the analyte peak area to IS peak area.

### 3.6. Statistical Analysis

All quantified data were scaled with unit variance scaling prior to analysis. To evaluate metabolic changes in feces from dogs fed the three different diets, principal component analyses (PCA) and partial least-squares discriminant analyses (PLS-DA) were conducted using SIMCA-P version 14.1 (Umetrics, Umeå, Sweden). As a general unsupervised statistical analysis, PCA projects the similarities and differences between numerous variables in a low dimensional plane [37]. In the score plot of a PCA, each point indicates an individual sample, and the distance between the points is associated with the correlations between samples. The supervised PLS-DA is generally applied to separate data according to known class memberships in the original dataset [38]. The quality of the PLS-DA model can be evaluated by the values of R^2^Y (percentage of variation explained by the model) and Q^2^ (predictive ability of the model). Values of R^2^Y > 0.7 and Q^2^ > 0.5 are considered “good.” Multiple PLS-DA models were acquired for samples at 8 weeks by comparing pairs of groups: control and QC, QCLB and control, and QCLB and QC. A comparison between the control and QC groups was conducted to evaluate possible metabolic changes related to the presence queso blanco cheese in the intestine. The comparison between the QCLB and control groups was performed to investigate the effects of queso blanco cheese containing *L. reuteri* KACC 92293 and *B. longum* KACC 91563 on intestinal health. Finally, the comparison between the QCLB and QC groups was carried out to evaluate metabolic changes induced by *L. reuteri* KACC 92293 and *B. longum* KACC 91563 when used as probiotics in queso blanco cheese. To identify the key variables that contributed to any separation of each pair of groups, a plot of the variables important in the projection (VIP) from the PLS-DA model was constructed. The VIP values in the plot represent relative values of contribution to the differences between groups [39]. The VIP plot constituted the components with VIP values greater than 1.0 as these values have an important influence on results of model.

To identify the significant differences (*p* < 0.05) after the feeding of probiotic cheese for 8 weeks, Student’s *t*-tests were run on the metabolites with VIP-value > 1.0. Box plots were constructed in GraphPad Prism 5 software (San Diego, CA, USA). A dendrogram was constructed using MetaboAnalyst 4.0 (www.metaboanalyst.ca), and Euclidean distance and Ward’s method were used, respectively, for distance measurements and clustering algorithm. To summarize the metabolic changes in feces related to intake of probiotic cheese at 8 weeks, a hierarchical clustering heat map with fold change (FC) analysis was used. The FC is the ratio of the average of each metabolite concentration in the treatment group divided by the average of each metabolite concentration in the control group. The FC was calculated using the formula log_2_FC. Multi-Experiment Viewer version 4.9.0 (MeV) was used to construct the hierarchical clustering heat map. The values of log_2_FC in three comparisons were determined as follow: QC Vs. control, QCLB Vs. QC, and QCLB Vs. control. The abundance of each metabolite was indicated by a range of −1.0 < log_2_FC < 1.0 with a specific color.

## 4. Conclusion

In this study, we applied metabolomics approaches to investigate the effects of the ingestion of queso blanco cheese containing *L. reuteri* KACC 92293 and *B. longum* KACC 91563 on the health of the intestines of dogs. Fecal metabolic profiling revealed the presence of 55 metabolites in the dog feces. Dynamic metabolic changes were confirmed by multivariate analysis. In the QCLB group, the levels of acetic acid, propionic acid, and GABA were higher compared to the control group, and xylose was detected at lower levels than in the QC group. Our results imply that the ingestion of queso blanco cheese with added *L. reuteri* KACC 92293 and *B. longum* KACC 91563 as probiotics positively changes the gut metabolites and intestinal environment in dogs. We identify propionic acid and xylose as candidate fecal biomarkers showing the effects of probiotic cheese in dogs for the first time. We propose that metabolomics approaches could be a useful tool for identifying characteristic phenotypes, such as potential biomarkers following probiotic cheese ingestion in humans and other animals. Probiotic cheese intake affects the compositions of gut microbiota, which can produce various metabolites. An interesting aspect for future study would be to relate the changes in the microbial community induced by probiotic cheese with the subsequent metabolomic changes. Therefore, we intend to explore the relationship between fecal microbiota and metabolites produced by them using 16S rRNA gene sequencing and metabolic profiling after feeding beagle dogs a diet containing probiotic queso blanco cheese.

## Supplementary Materials

The following are available online at https://www.mdpi.com/2218-1989/10/8/305/s1. Figure S1. Dendrogram of control, C, and QCLB groups associated with different genders after 8 weeks of feeding. Table S1: Volatile fatty acids and indolic compound identified by GC–MS in the feces of dogs. Table S2: Long chain fatty acids identified by GC–FID in the feces of dogs. Table S3: Hydrophilic compounds identified by GC–MS in the feces of dogs.

## References

[1] Gibson G.R., Hutkins R., Sanders M.E., Prescott S.L., Reimer R.A., Salminen S.J., Scott K., Stanton C., Swanson K.S., Cani P.D. (2017). Expert consensus document: The international scientific association for probiotics and prebiotics (isapp) consensus statement on the definition and scope of prebiotics. Nat. Rev. Gastroenterol. Hepatol..

[2] Linares D.M., Gómez C., Renes E., Fresno J.M., Tornadijo M.E., Ross R.P., Stanton C. (2017). Lactic acid bacteria and bifidobacteria with potential to design natural biofunctional health–promoting dairy foods. Front. Microbiol..

[3] Mu Q., Tavella V.J., Luo X.M. (2018). Role of *Lactobacillus reuteri* in Human Health and Dis. Front. Microbiol..

[4] Linares D.M., Ross P., Stanton C. (2016). Beneficial microbes: The pharmacy in the gut. Bioengineered.

[5] Tenorio–Jiménez C., Martínez-Ramírez M.J., Tercero-Lozano M., Arraiza–Irigoyen C., Del Castillo-Codes I., Olza J., Plaza-Díaz J., Fontana L., Migueles J.H., Olivares M. (2018). Evaluation of the effect of *Lactobacillus reuteri* V3401 on biomarkers of inflammation, cardiovascular risk and liver steatosis in obese adults with metabolic syndrome: A randomized clinical trial (PROSIR). BMC Complement. Altern. Med..

[6] Wu R.Y., Jeffrey M.P., Johnson–Henry K.C., Green–Johnson J.M., Sherman P.M. (2017). Impact of prebiotics, probiotics, and gut derived metabolites on host immunity. Lympho Sign J..

[7] Elian S.D.A., Souza E.L.S., Vieira A.T., Teixeira M.M., Arantes R.M.E., Nicoli J.R., Martins F.S. (2015). *Bifidobacterium Longum* Subsp. Infantis BB-02 Attenuates Acute Murine Experimental Model of Inflammatory Bowel Disease. Benef. Microbes.

[8] Tomosada Y., Villena J., Murata K., Chiba E., Shimazu T., Aso H., Iwabuchi N., Xiao J.-Z., Saito T., Kitazawa H. (2013). Immunoregulatory effect of bifidobacteria strains in porcine intestinal epithelial cells through modulation of ubiquitin–editing enzyme A20 expression. PLOS ONE.

[9] Mathipa M.G., Thantsha M.S. (2017). Probiotic engineering: Towards development of robust probiotic strains with enhanced functional properties and for targeted control of enteric pathogens. Gut Pathog..

[10] Karimi R., Mortazavian A.M., Karami M. (2012). Incorporation of *Lactobacillus casei* in Iranian ultrafiltered Feta cheese made by partial replacement of NaCl with KCl. J. Dairy Sci..

[11] Lahtinen S.J., Forssten S., Aakko J., Granlund L., Rautonen N., Salminen S., Viitanen M., Ouwehand A.C. (2012). Probiotic cheese containing *Lactobacillus rhamnosus* HN001 and *Lactobacillus acidophilus* NCFM^®^ modifies subpopulations of fecal lactobacilli and *Clostridium difficile* in the elderly. Age.

[12] Coelho L.P., Kultima J.R., Costea P.I., Fournier C., Pan Y., Czarnecki-Maulden G., Hayward M.R., Forslund S.K., Schmidt T.S.B., Descombes P. (2018). Similarity of the dog and human gut microbiomes in gene content and response to diet. Microbiome.

[13] Xu H., Zhao F., Hou Q., Huang W., Liu Y., Zhang H., Sun Z. (2019). Metagenomic analysis revealed beneficial effects of probiotics in improving the composition and function of the gut microbiota in dogs with diarrhoea. Food Funct..

[14] Chung H.J., Sim J.H., Min T.S., Choi H.K. (2018). Metabolomics and Lipidomics Approaches in the Science of Probiotics: A Review. J. Med. Food.

[15] Tarbah F.A., Mahler H., Temme O., Daldrup T. (2001). An analytical method for the rapid screening of organophosphate pesticides in human biological samples and foodstuffs. Forensic Sci. Int..

[16] Lotti C., Rubert J., Fava F., Tuohy K., Mattivi F., Vrhovsek U. (2017). Development of a fast and cost–effective gas chromatography–mass spectrometry method for the quantification of short–chain and medium–chain fatty acids in human biofluids. Anal. Bioanal. Chem..

[17] Zhang H., Wang Z., Liu O. (2015). Development and validation of a GC–FID method for quantitative analysis of oleic acid and related fatty acids. J. Pharm. Anal..

[18] Griffiths W.J., Koal T., Wang Y., Kohl M., Enot D.P., Deigner H.P. (2010). Targeted metabolomics for biomarker discovery. Angew. Chem. Int. Ed..

[19] Wishart D.S. (2008). Metabolomics: Applications to food science and nutrition research. Trends Food Sci. Technol..

[20] Mozzi F., Ortiz M.E., Bleckwedel J., De Vuyst L., Pescuma M. (2013). Metabolomics as a tool for the comprehensive understanding of fermented and functional foods with lactic acid bacteria. Food Res. Int..

[21] Park H.-E., Kim Y.J., Do K.-H., Kim J.K., Ham J.-S., Lee W.-K. (2018). Effects of queso blanco cheese containing Bifidobacterium longum KACC 91563 on the intestinal microbiota and short chain fatty acid in healthy companion dogs. Korean J. Food Sci. Anim. Resour..

[22] Park H.E., Kim Y.J., Kim M., Kim H., Do K.H., Kim J.K., Ham J.-S., Lee W.K. (2020). Effects of Queso Blanco cheese containing *Bifidobacterium longum* KACC 91563 on fecal microbiota, metabolite and serum cytokine in healthy beagle dogs. Anaerobe.

[23] Wong J.M., De Souza R., Kendall C.W., Emam A., Jenkins D.J. (2006). Colonic health: Fermentation and short chain fatty acids. J. Clin. Gastroenterol..

[24] Parnell-Clunies E.M., Irvine D., Bullock D. (1985). Composition and yield studies for Queso Blanco made in pilot plant and commercial trials with dilute acidulant solutions. J. Dairy Sci..

[25] Fukuda S., Toh H., Taylor T.D., Ohno H., Hattori M. (2012). Acetate-producing bifidobacteria protect the host from enteropathogenic infection via carbohydrate transporters. Gut Microbes.

[26] da Cruz A.G., Buriti F.C.A., de Souza C.H.B., Faria J.A.F., Saad S.M.I. (2009). Probiotic cheese: Health benefits, technological and stability aspects. Trends Food Sci. Technol..

[27] Pokusaeva K., Johnson C., Luk B., Uribe G., Fu Y., Oezguen N., Matsunami R., Lugo M., Major A., Mori-Akiyama Y. (2017). GABA–producing *Bifidobacterium dentium* modulates visceral sensitivity in the intestine. Neurogastroenterol. Motil..

[28] Siragusa S., De Angelis M., Di Cagno R., Rizzello C.G., Coda R., Gobbetti M. (2007). Synthesis of γ–aminobutyric acid by lactic acid bacteria isolated from a variety of Italian cheeses. Appl. Environ. Microbiol..

[29] Thierry A., Maillard M.-B. (2002). Production of cheese flavour compounds derived from amino acid catabolism by *Propionibacterium freudenreichii*. Lait.

[30] Mäkeläinen H., Forssten S., Olli K., Granlund L., Rautonen N., Ouwehand A. (2009). Probiotic lactobacilli in a semi-soft cheese survive in the simulated human gastrointestinal tract. Int. Dairy J..

[31] Elsden S.R., Hilton M.G. (1978). Volatile acid production from threonine, valine, leucine and isoleucine by clostridia. Arch. Microbiol..

[32] Liu H., Hou C., Wang G., Jia H., Yu H., Zeng X., Thacker P.A., Zhang G., Qiao S. (2017). *Lactobacillus reuteri* I5007 modulates intestinal host defense peptide expression in the model of IPEC–J2 cells and neonatal piglets. Nutrients.

[33] Staudigl P., Haltrich D., Peterbauer C.K. (2014). L-Arabinose isomerase and D-xylose isomerase from Lactobacillus reuteri: Characterization, coexpression in the food grade host *Lactobacillus plantarum*, and application in the conversion of D–galactose and D-glucose. J. Agric. Food Chem..

[34] Yan S., Zhao G., Liu X., Zhao J., Zhang H., Chen W. (2017). Production of exopolysaccharide by *Bifidobacterium longum* isolated from elderly and infant feces and analysis of priming glycosyltransferase genes. RSC Adv..

[35] Ham J.S., Jeong S.G., Noh Y.B., Shin J.H., Han G.S., Chae H.S., Yoo Y.M., Ahn J.N., Lee J.W., Jo C. (2007). Effects of gamma irradiation on Queso Blanco cheese. Korean J. Dairy Sci. Technol..

[36] Kim Y.J., Kim J.G., Lee W.-K., So K.M., Kim J.K. (2019). Trial data of the anti–obesity potential of a high resistant starch diet for canines using Dodamssal rice and the identification of discriminating markers in feces for metabolic profiling. Metabolomics.

[37] Zheng S., Yu M., Lu X., Huo T., Ge L., Yang J., Wu C., Li F. (2010). Urinary metabonomic study on biochemical changes in chronic unpredictable mild stress model of depression. Clin. Chim. Acta..

[38] Eriksson L., Byrne T., Johansson E., Trygg J., Vikström C. (2013). Multi–and Megavariate Data Analysis Basic Principles and Applications.

[39] Oberg J., Spenger C., Wang F.-H., Andersson A., Westman E., Skoglund P., Sunnemark D., Norinder U., Klason T., Wahlund L.-O. (2008). Age related changes in brain metabolites observed by 1H MRS in APP/PS1 mice. Neurobiol. Aging.

