# Supplementary material for: Investigations on Metabolic Changes in Beagle Dogs Fed Probiotic Queso Blanco Cheese and Identification of Candidate Probiotic Fecal Biomarkers Using Metabolomics Approaches
Source: Metabolites. 2020 Jul 25;10(8):305. doi: 10.3390/metabo10080305 (PMC7464839; doi:10.3390/metabo10080305)
Supplement: Supplementary file 1 [file metabolites-10-00305-s001.pdf]

Investigations on Metabolic Changes in Beagle Dog fed Probiotic Queso Blanco Cheese and Identification of Candidate Probiotic Fecal Biomarkers using Metabolomics Approaches

Ye Jin Kim [1,†], Ho-Eun Park [2], Wan-Kyu Lee [2], Jun-Sang Ham [3], Sang Un Park [4,†], Jae Geun Kim [1,*], Kyung-Hoan Im [1] and Jae Kwang Kim [1,*]

[1] Division of Life Sciences, College of Life Sciences and Bioengineering, Incheon National University, Yeonsugu, Incheon 22012, Republic of Korea
[2] College of Veterinary Medicine, Chungbuk National University, Cheongju 28644, Republic of Korea;
[3] Animal Products Development and Utilization Division, National Institute of Animal Science, Wanju 55365, Republic of Korea
[4] Department of Crop Science, Chungnam National University, 99 Daehak-ro, Yuseong-gu, Daejeon 34134, Republic of Korea
* Correspondence: jgkim@inu.ac.kr (J.G.K.); Tel.: +82-32-835-8256; kjkpj@inu.ac.kr (J.K.K.); Tel.: +82-32-835-8241
† These authors contributed equally to this work.

Supplementary File

Figure S1. Dendrogram of Control, QC, and QCLB groups associated with different genders after 8 weeks of feeding

Table S1. Volatile fatty acids and indolic compound identified by GC-MS in the feces of dogs.

Table S2. Long chain fatty acids identified by GC-FID in the feces of dogs.

Table S3. Hydrophilic compounds identified by GC-MS in the feces of dogs.

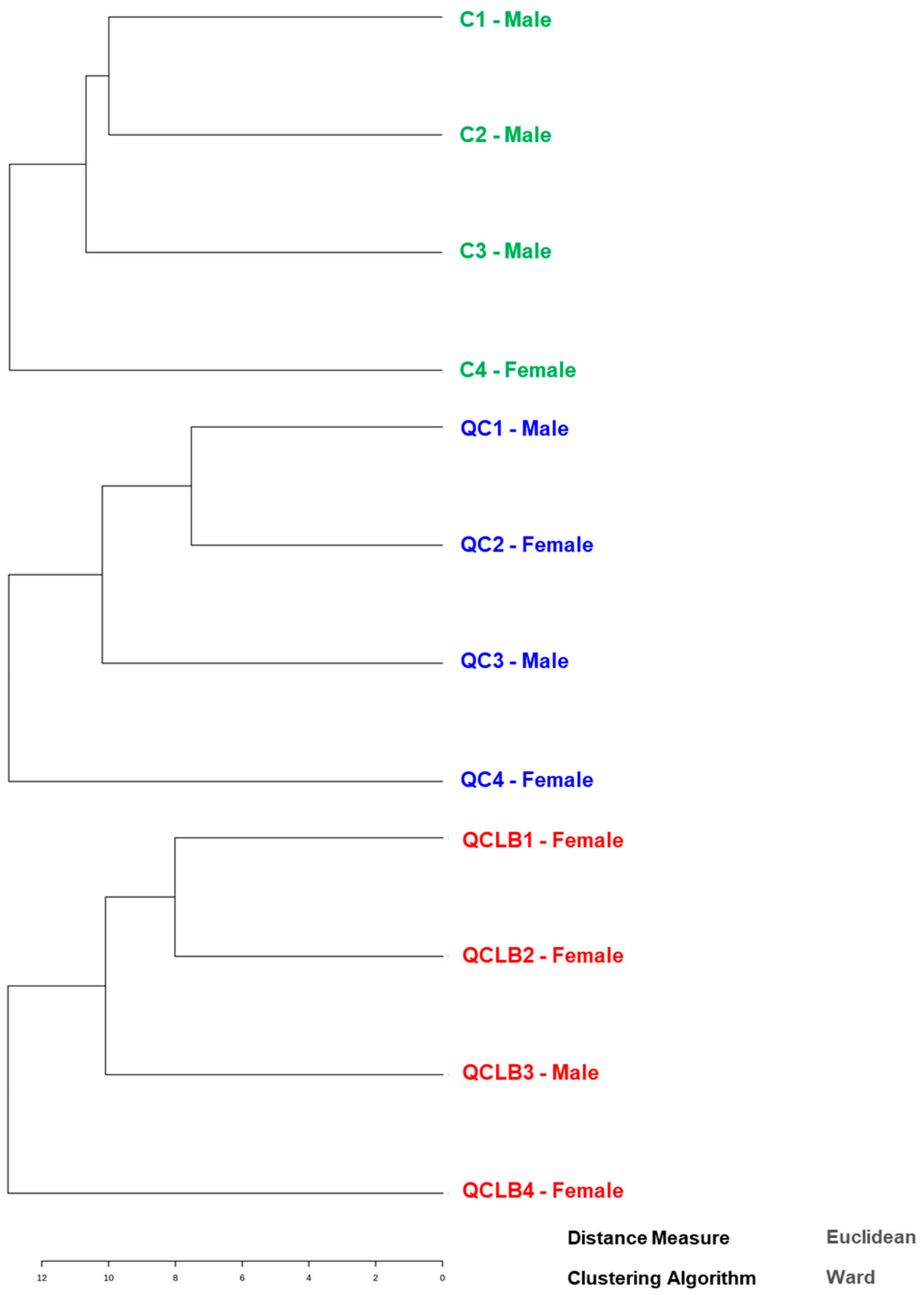

Figure S1. Dendrogram of Control, QC, and QCLB groups associated with different genders after 8 weeks of feeding. C, Control; regular diet and queso blanco cheese; QCLB, regular diet and queso blanco cheese with added *L. reuteri* KACC 92293 and *B. longum* KACC 91563.

Table S1. Volatile fatty acids and indolic compound identified by GC-MS in the feces of dogs.

Compound	RT[1]	RRT[2]	Quantitation ion[3] m/z (%)	Other characteristic ions m/z (%)
Acetic acid	2.445	0.400	43 (100)	45 (82), 60 (57)
Propionic acid	3.744	0.613	74 (100)	45 (86)
Butyric acid	4.790	0.785	60 (100)	41 (28), 73 (28)
Iso valeric acid	5.335	0.874	60 (100)	41 (45), 43 (53)
Valeric acid	5.662	0.927	60 (100)	73 (35)
2-Methylvaleric acid (IS)[4]	6.105	1.000	74 (100)	43 (53)
Indole	8.040	1.317	117 (100)	90 (37)

[1] Retention time (min)

[2] Relative retention times (retention time of analyte/retention time of 2-methylvaleric acid)

[3] Retention index

[4] Internal standard

Table S2. Long chain fatty acids identified by GC-FID in the feces of dogs.

Compound	C:D[1]	RT[2]	RRT[3]
Lauric acid	C12:0	6.149	0.759
Myristic acid	C14:0	7.488	0.925
Pentadecanoic acid (IS)[4]	C15:0	8.097	1.000
Palmitic acid	C16:0	8.660	1.070
Palmitoleic acid	C16:1	8.813	1.088
Stearic acid	C18:0	9.944	1.228
Oleic acid	C18:1	10.089	1.246
Linoleic acid	C18:2	10.419	1.287
α-Linolenic acid	C18:3n3	10.912	1.348
Arachidic acid	C20:0	11.542	1.425
Dihomo-γ-linolenic acid	C20:3n6	12.440	1.536
Arachidonic acid	C20:4n6	12.667	1.564
Eicosapentaenoic acid	C20:5n3	13.358	1.650
Behenic acid	C22:0	13.601	1.680
Docosapentaenoic acid	C22:5n3	15.990	1.975
Docosahexaenoic acid	C22:6n3	16.255	2.008

[1] Total amount of Carbon atoms (C) of the fatty acid:the number of Double (unsaturated) bonds (D)

[2] Retention time (min)

[3] Relative retention times (retention time of analyte/retention time of pentadecanoic acid)

[4] Internal standard

Table S3. Hydrophilic compounds identified by GC-MS in the feces of dogs.

Compound	RT[1]	RRT[2]	RI[3]	Quantitation ion[4] m/z (%)	Other characteristic ion m/z (%)
Pyruvic acid	8.127	0.429	1047	174 (100)	158 (7)
Lactic acid	8.397	0.443	1061	219 (27)	191 (100)
Glycolic acid	8.649	0.457	1074	205 (50)	177 (100)
Alanine	9.258	0.489	1106	190 (23)	147 (100)
Valine	11.281	0.596	1222	218 (16)	144 (100)
Leucine	12.184	0.643	1278	232 (100)	218 (66)
Glycerol	12.200	0.644	1279	218 (39)	205 (100)
Phosphoric acid	12.216	0.645	1280	314 (19)	299 (100)
Isoleucine	12.553	0.663	1301	232 (26)	218 (100)
Nicotinic acid	12.597	0.665	1304	180 (100)	-
Proline	12.627	0.667	1306	216 (55)	147 (100)
Succinic acid	12.730	0.672	1313	247 (100)	172 (42)
Glycine	12.774	0.675	1316	248 (24)	174 (100)
Glyceric acid	13.114	0.693	1339	292 (71)	189 (100)
Fumaric acid	13.203	0.697	1345	245 (100)	217 (5)
Serine	13.528	0.714	1367	306 (49)	278 (100)
Threonine	13.956	0.737	1396	291 (41)	218 (100)
β-Alanine	14.508	0.766	1436	174 (100)	-
Malic acid	15.329	0.810	1496	233 (100)	189 (56)
Aspartic acid	15.754	0.832	1529	334 (43)	306 (100)
Methionine	15.766	0.833	1530	293 (100)	250 (81)
4-Aminobutyric acid	15.906	0.840	1541	304 (32)	174 (100)
Glutamic acid	16.995	0.898	1628	363 (56)	348 (100)
Phenylalanine	17.163	0.906	1642	218 (100)	192 (69)
Xylose	17.571	0.928	1676	307 (100)	277 (20)
Glutamine	18.821	0.994	1785	362 (43)	347 (100)
L-2-Chlorophenylalanine (IS)[5]	18.935	1.000	1795	218 (100)	-
Mannose	19.763	1.044	1872	217 (22)	204 (100)
Quinic acid	19.892	1.051	1884	345 (100)	-
Fructose	20.105	1.062	1904	307 (100)	277 (20)
Glucose 1	20.372	1.076	1930	319 (100)	205 (62)
Glucose 2	20.555	1.086	1951	319 (100)	205 (62)

Inositol	22.359	1.181	2132	318 (54)	305 (100)
Tryptophan	23.447	1.238	2251	405 (100)	377 (88)
Sucrose	27.280	1.441	2705	437 (20)	361 (100)
Trehalose	28.341	1.497	2812	361 (100)	271 (12)

[1] Retention time (min)

[2] Relative retention times (retention time of analyte/retention time of 2-methylvaleric acid)

[3] Retention index

[4] Specific mass ion used for quantitation

[5] Internal standard
